# Victimization and Perpetration Experiences of Adults With Autism

**DOI:** 10.3389/fpsyt.2018.00203

**Published:** 2018-05-25

**Authors:** Jonathan A. Weiss, Michelle A. Fardella

**Affiliations:** Department of Psychology, York University, Toronto, ON, Canada

**Keywords:** autism, emotion regulation, social skills, victimization, perpetration, bullying, adults, maltreatment

## Abstract

This study aimed to describe the self-reported experiences of childhood and adult victimization and perpetration in adults with autism spectrum conditions (ASC) compared to a matched sample, and how victimization and perpetration are associated with autism-related difficulties. Forty-five adults with ASC and 42 adults without ASC completed questionnaires regarding violence victimization and perpetration, emotion regulation, and sociocommunicative competence. Participants with ASC reported experiencing, as children, more overall victimization; specifically, more property crime, maltreatment, teasing/emotional bullying, and sexual assault by peers, compared to participants without ASC. Participants with ASC also reported experiencing more teasing/emotional bullying in adulthood and greater sexual contact victimization. No significant differences were found between groups on perpetration. Sociocommunicative ability and emotion regulation deficits did not explain the heightened risk for victimization. Individuals with ASC have an increased vulnerability to violence victimization, which speaks to the need for interventions, and proactive prevention strategies.

## Introduction

Adults with autism spectrum conditions (ASC) may be at considerable risk for interpersonal violence victimization, which refers to violence and abuse that occurs between people, including child maltreatment, intimate partner violence, adolescent dating violence, and bullying (1). Individuals with ASC have a number of impairments in social communication and social interaction across multiple contexts, and exhibit restricted interests and/or repetitive body movements and behaviors (2). The current estimated prevalence of ASC is ~1 in 68, with ~44% having average to above average intellectual abilities (3, 4).

There is a paucity of research examining discrete experiences of interpersonal violence in those with ASC, although what does exist points to an increased risk for child maltreatment, bullying, and sexual victimization (5–7). In children, having an autism diagnosis is associated with an increased chance of physical, emotional, or sexual abuse compared to peers without disabilities (8). More recent research interviewing 182 parents of children with ASC found high rates of reported physical abuse (18.5%), sexual abuse (12.2%), or both kinds (4.4%), though no information on the sources of this abuse was noted (7). Studies have also found high rates of peer victimization in children [65–77%; (6, 9)]. Studies of adults with ASC have largely focused exclusively on sexual victimization. In a college sample, students with ASC were twice as likely to report unwanted sexual contact compared to students without ASC (10). In an online survey, 70% of adults with ASC reported experiencing some form of sexual victimization after age 14 and into adulthood, compared to 45% of those without ASC (5). Authors have suggested that increased risks of bullying, physical, and emotional abuse may also be present in adults with ASC due to heightened social vulnerability (11, 12).

Research has begun to move from an understanding of experiences of interpersonal violence in isolation to understanding the co-occurrence and interconnections between experiences of interpersonal violence, known as polyvictimization, and polyperpetration (13). Too often forms of violence are studied in isolation, and some authors state that focusing on specific forms in isolation may mask the important information that would be gained by studying the complex, varied patterns of traumas (14). Research has yet to examine the broader interpersonal violence experiences of adults with ASC beyond sexual violence victimization, or to look at interpersonal violence perpetration in adults in the community, though what does exist on this latter question suggests no clear association between ASC and violent crime (15–17). Additional research is needed to understand the context of violence across a number of different kinds of acts in adults with ASC.

It is critical to understand the mechanisms that are associated with heightened risk for interpersonal violence (18). Deficits in sociocommunicative competence may be a particular set of risk factors for violence victimization and perpetration in adults with ASC (19, 20). It is well known that individuals with ASC can have challenges with social reasoning, are literal thinkers, and may miss contextual cues (21, 22), and authors have suggested that such sociocommunicative difficulties may be related to an increased risk of sexual abuse (23) and bullying in children with ASC (6). With regard to perpetration, social naivety and misinterpretation of social cues may inadvertently lead to criminal behavior in individuals with ASC, though not specifically to interpersonal violence (24–26). For instance, authors have noted that individuals with ASC inadvertently engage in stalking behaviors when they seek out contact with others for friendship or intimacy [e.g., (27–29)]. No study has tested whether sociocommunicative difficulties explain an increased risk of violence for adults with ASC.

Emotion regulation deficits have also been linked with violence victimization and perpetration in adults in general [e.g., (30)], and may be a particularly salient factor for adults with ASC. In children and adults without ASC, maladaptive emotion regulation is a risk factor for chronic victimization (31, 32). For perpetration, the ability to regulate one's negative emotions may be a factor that helps individuals refrain from initiating violence (33). While difficulties in emotion regulation, emotional expression, and emotion processing have been widely discussed in the ASC literature (34), its link to violence in this population has only been briefly explored, with one study reporting an association among emotion dysregulation and bullying perpetration and victimization in youth (35).

The negative effects of violence are well known in the non-ASC literature (36), and additional efforts to understand the prevalence, characteristics, and causes in adults with ASC are needed. The current study aimed to identify (1) patterns of violence victimization and perpetration in adults with and without ASC across many types, (2) differences in self-reported polyvictimization and polyperpetration, and (3) whether impairments in the areas of sociocommunicative competence and emotion regulation mediate the expected higher rates of violence victimization and perpetration. Self-report was used to gain a reliable estimate of violence victimization and perpetration experiences in adults with ASC living in the community.

## Methods

### Recruitment

Participants with ASC were recruited through study notices distributed by community-based programs and organizations that support those with ASC, online ASC communities, several colleges/universities academic support services, and from study participants to others at their discretion. The comparison group was recruited through postings within the University setting and on community message boards. Advertisements indicated that this was a research project on interpersonal violence in adults that aimed to understand the experiences of interpersonal violence, and that any adult could participate, even if they did not experience violence themselves. Identical recruitment and consent materials were used for both groups. Eligible participants with ASC were required to (a) have a diagnosis of an ASC (e.g., Autism, Asperger Syndrome, Autism Spectrum Disorder, PDD-NOS) according to self-report, which was verified by administering the Autism Diagnostic Observation Schedule-−2nd Edition (37), (b) be 18 years of age or older, and (c) have an estimated IQ above 80 on the Wechsler Abbreviated Scale of Intelligence (38). Participants without ASC had to meet criterion b and c. Equal numbers of men and women with ASC responded to the study advertisements.

### Participants

The sample included 45 adults with ASC between 18-53 years of age (*M* = 30.00, *SD* = 1.48) and 42 adults without ASC, matched on mean chronological age, between 19 and 54 years (*M* = 32.12, *SD* = 8.62). Groups did not significantly differ with respect to the percentage of men (42.5% ASC; 50% non-ASC) or on self-identified minority status (15.6% ASC; 31% non-ASC). Participants were also similar in IQ estimates (non-ASC *M* = 113.33, *SD* = 16.10, Range 87–146; ASC *M* = 110.22, *SD* = 13.19, Range 81–134; *t*_(85)_ = −0.98, *p* = 0.36), and in the percentage who obtained at least a college degree (85% ASC; 95% non-ASC). All participants lived in the Greater Toronto Area. Participants in the ASC group reported a diagnosis of ASC and met the clinical cut-off on the ADOS-2 Module 4 (37). Participants without ASC reported that they did not identify with being on the autism spectrum and had never received a diagnosis of an ASC (e.g., autism, Asperger's Syndrome, etc…).

### Measures

#### Autism diagnostic observation scale- 2nd edition (ADOS-2)

The ADOS-2 (37) is a semi-structured observational measure that examines social and communicative behaviors, and was used to verify ASC status for the ASC group. The ADOS has been found to have good test-retest reliability and excellent internal consistency (37).

#### Wechsler abbreviated scale of intelligence (WASI)

The four-subset WASI (38) was administered to obtain a general estimate of intellectual functioning (Full Scale IQ). This measure has been shown to have adequate to high test-retest reliability (*r* = 0.72–0.95) depending on the subtest, and high internal consistency across groups and subtests (Cronbach's alpha = 0.87–0.98). The WASI has been used in adults with ASC as a brief measure of IQ (39).

#### Juvenile victimization questionnaire-adult retrospective questionnaire (JVQ-AR)

The JVQ-AR was used as a measure of childhood victimization, adult victimization, and adult perpetration. The original child victimization version is a 34-item self-report questionnaire that collects information on several forms of childhood victimization (40). The questionnaire assesses the frequency of 34 discrete forms of childhood victimization, scored as a dichotomy (1 = *experienced*; 0 = not *experienced*). For *childhood victimization*, participants are asked about any experiences from birth up until the 18th birthday (0 through 17 years 12 months). The 34 questions fall within six categories: property crime, physical assault, child maltreatment, peer/sibling victimization, sexual victimization, and witnessed/indirect victimization. For *adult victimization*, a modified version of JVQ-AR was used where participants reported on any of 29 victimization experiences across the same 6 categories, which occurred from their 18th birthday onward. Items that pertained to childhood experiences were removed. Scores are provided for each individual item and each aggregate category. Polyvictimization was computed by summing the endorsed victimization items, with scores ranging from 0 to 34 for childhood victimization, and 0 to 29 for adult victimization [as recommended by (41)], with higher scores indicating a greater number of discrete victimization experiences.

For *adult perpetration*, a modified version of the JVQ-AR was used where participants were asked about perpetration experiences that occurred from age 18 on. Items pertaining to witnessing violence (e.g., witnessing domestic violence) and child maltreatment (e.g., being bullied by peers) were removed, since the focus of this questionnaire was adulthood and perpetration experiences (i.e., acts committed by the individual during adulthood). Polyperpetration was computed by summing the endorsed perpetration items, with scores ranging from 0 to 19, with higher scores indicating a greater number of discrete acts of violence.

#### Difficulties in emotion regulation scale (DERS)

The DERS (42) is a 36-item self-report measure of emotion regulation ability. Subscales assess six dimensions of difficulties: Nonacceptance, Goals, Impulse, Awareness, Strategies, and Clarity. Participants rate how often statements apply to them on a Likert scale with answer categories: 1 = *almost never* to 5 = *almost always*. An overall score was used for the current study. Higher scores indicate greater difficulty with emotion regulation. The DERS has been shown to have good internal consistency, test-retest reliability, and construct validity (42–44), and was recently used in a sample of young adults with ASC (45). Internal consistency for the DERS across the whole sample and individual groups demonstrated good to excellent reliability (whole sample α = 0.95, ASC group α = 0.89, no ASC group α = 0.94).

#### Multidimensional social competence scale (MSCS)

Sociocommunicative competence was measured utilizing the self-report version of the MSCS (46). The MSCS is designed to assess social competence among adolescents and adults with ASC. Psychometric evidence provided preliminary support for the reliability and validity of the scale [Cronbach's alpha reliabilities for domain, subscale, and total scores were all above 0.84; (46)]. The MSCS measures seven domains of social competence: social motivation, social inferencing, demonstrating empathic concern, social knowledge, verbal conversation skills, nonverbal sending skills, and emotion regulation. Participants rated how statements applied to them, where 1 = *not true or almost never true*, to 5 = *very true or almost always true*. An overall score was used for the current study, without including emotion regulation (given the use of the DERS). Overall Cronbach's alpha within both groups demonstrated excellent internal consistency (no ASC group α = 0.95, ASC group α = 0.93).

### Procedure

All participants met in person with a trained graduate student. Informed consent was obtained, IQ was assessed, and for those with ASC, the ADOS-2 was completed. Participants were then provided with a laptop computer to complete questionnaires on the online Qualtrics data system (www.qualtrics.com). The University ethics board approved this research. Participants with and without ASC received a gift card to an online retailer for their participation. The informed consent articulated the limits of confidentiality and that participants may have experienced feelings of discomfort generated by the content of the questions asked. A list of support resources were provided to all participants, and they were informed that if they experienced any emotional distress and wanted to speak with a counselor, the researcher would facilitate. One participant with ASC requested this information.

### Data analysis

Chi-square analyses and odds ratios were used to examine whether there were differences in the self-report of victimization and perpetration between groups. Due to non-normal data, the Mann Whitney test was calculated to compare groups on self-reported breadth of victimization and perpetration, on sociocommunicative competence, and emotion regulation abilities. Preliminary analyses revealed no differences when comparing men to women in either of the two groups (e.g., men with ASC vs. women with ASC, or men without ASC to women without ASC) on overall polyvictimization or polyperpetration, and on aggregate scores, within either the ASC group or non-ASC group (all *p*'s >.10).

To establish whether self-reported polyvictimization and polyperpetration experiences would be mediated by deficits in sociocommunicative competence and emotion regulation, a test of multiple mediation was run using SPSS INDIRECT macro script for testing multiple mediator models with bootstrapping (47). Given the large age range and concerns that men and women could differ in terms of their experiences of victimization or perpetration, all mediation analyses entered age and sex as control variables (the same analyses were run without these controls and no differences emerged in the pattern of results).

## Results

### Childhood victimization

As shown in Table 1, during their childhood, participants with ASC were 6.7 times more likely to report experiencing a form of property crime, largely the result of being more likely to have been robbed than peers without ASC. Those with ASC were 4 times more likely to report experiencing a form of child maltreatment, including physical abuse, and psychological or emotional abuse from adults, 27.1 times more likely to endorse teasing from peers, 3.7 times more likely to endorse bullying from peers, and 7.3 times more likely to endorse sexual assault by a peer, compared to adults without ASC. Participants without ASC were 4.4 times more likely to endorse having sexual relations with someone over 18 than participants with ASC. Participants with ASC reported significantly higher polyvictimization than those without ASC (*U* = 1204, *p* = 0.03; ASC *M* = 12.62, *SD* = 5.45; no ASC *M* = 10.05, *SD* = 7.12).

**Table 1:** Frequency Table for the 34 types of childhood victimization on the JVQ-AR as reported by adults with and without ASC.

**Victimization Type**	**ASC *n* (%)**	**No ASC *n* (%)**	**Chi-square/Fisher's exact**
34 types of victimization, at least one type	45 (100)	41 (97.6)	χ2_(1)_ = 1.08, *p* = 0.30
Property Crime aggregate (at least one type)	43 (95.6)	32 (76.2)	Fisher's exact *p* = 0.01; OR = 6.7
Robbery	40 (90.9)	22 (47.6)	Fisher's exact *p* < 0.0001; OR = 9.1
Theft	31 (68.9)	25 (59.5)	χ2_(1)_ = 0.83, *p* = 0.36
Vandalism	30 (68.2)	23 (54.8)	χ2_(1)_ = 1.64, *p* = 0.20
Physical Assault aggregate (at least one type)	43 (95.6)	37 (88.1)	Fisher's exact *p* = 0.26
Assault with a weapon	24 (53.5)	19 (45.2)	χ2_(1)_ = 0.57, *p* = 0.45
Assault without a weapon	37 (82.2)	28 (66.7)	χ2_(1)_ = 2.78, *p* = 0.09; OR = 2.31
Attempted assault	22 (48.9)	13 (31)	χ2_(1)_ = 2.91, *p* = 0.09; OR = 2.13
Kidnap, attempted, or completed	5 (11.1)	3 (7.1)	Fisher's exact *p* = 0.71
Bias attack	7 (15.6)	8 (19)	χ2_(1)_ = 0.19, *p* = 0.67
Physical abuse (not spanking)	26 (57.8)	11 (26.2)	χ2_(1)_ = 8.87, *p* = 0.003; OR = 3.9
Assault by group or gang of peers	23 (51.1)	14 (33.3)	χ2_(1)_ = 2.81, *p* = 0.09; OR = 2.09
Peer/sibling assault	35 (77.8)	32 (76.2)	χ2_(1)_ = 0.03, *p* = 0.86
Genital assault	21 (46.7)	15 (35.7)	χ2_(1)_ = 1.07, *p* = 0.30
Dating violence	3 (6.7)	5 (11.9)	Fisher's exact *p* = 0.48
Child maltreatment	36 (80)	21 (51)	χ2_(1)_ = 8.65, *p* = 0.003; OR = 4.0
Physical abuse (not spanking)	26 (57.8)	11 (26.2)	χ2_(1)_ = 8.87, *p* = 0.003; OR = 3.9
Psychological or emotional abuse	28 (62.2)	15 (35.7)	χ2_(1)_ = 6.11, *p* = 0.01; OR = 3.4
Neglect	9 (20)	6 (14.3)	χ2_(1)_ = 49, *p* = 0.48
Custodial interference or family abduction	5 (11.1)	5 (11.9)	Fisher's exact *p* = 1.0
Peer/sibling victimization aggregate (at least one type**)**	44 (97.8)	36 (85.7)	Fisher's exact *p* = 0.05; OR = 7.33
Assault by group or gang	23 (51.1)	14 (33.3)	χ2_(1)_ = 2.81, *p* = 0.09; OR = 2.09
Peer/sibling assault	35 (77.8)	32 (76.2)	χ2_(1)_ = 0.03, *p* = 0.86
Genital assault	21 (46.7)	15 (35.7)	χ2_(1)_ = 1.07, *p* = 0.30
Bullying	34 (75.6)	19 (45.2)	χ2_(1)_ = 8.39, *p* = 0.004; OR = 3.7
Teasing, emotional bullying	44 (97.8)	26 (61.9)	Fisher's exact *p* < 0.001;OR = 27.1
Dating violence	3 (6.7)	5 (11.9)	Fisher's exact *p* = 0.48s
Witnessed/indirect victimization aggregate (at least one type)	35 (77.8)	31 (73.8)	χ2_(1)_ = 0.19, *p* = 0.67
Witness domestic violence	8 (17.8)	9 (21.4)	χ2_(1)_ = 0.18, *p* = 0.67
Witness physical abuse	10 (22.2)	8 (22.2)	χ2_(1)_ = 0.10, *p* = 0.76
Witness assault with a weapon	17 (37.8)	18 (42.9)	χ2_(1)_ = 0.23, *p* = 0.63
Witness assault without a weapon	26 (59.1)	25 (61)	χ2_(1)_ = 0.03, *p* = 0.86
Household theft	22 (50)	16 (39)	χ2_(1)_ = 1.03, *p* = 0.31
Someone close murdered	0 (0)	4 (9.5)	Fisher's exact *p* = 0.05
Witness murder	1 (2.3)	2 (4.9)	Fisher's exact *p* = 0.61
Exposure to shooting, bombs, riots	4 (9.1)	10 (23.8)	Fisher's exact *p* = 0.08; OR = 3.13
Sexual victimization aggregate (at least one type)	25 (55.6)	21 (50)	χ2_(1)_ = 0.27, *p* = 0.60
Sexual assault, known adult	7 (15.6)	7 (16.7)	χ2_(1)_ = 0.02, *p* = 0.89
Sexual assault, unknown adult	3 (6.7)	2 (4.8)	Fisher's exact *p* = 1.00
Sexual assault, with peer	12 (26.7)	2 (4.8)	Fisher's exact *p* = 0.007; OR = 7.3
Rape, attempted or completed	6 (13.3)	5 (11.9)	Fisher's exact *p* = 1.00
Flashing or sexual exposure	9 (20)	6 (14.3)	χ2_(1)_ = 0.50, *p* = 0.48
Sexual harassment	16 (35.6)	11 (26.2)	χ2_(1)_ = 0.89, *p* = 0.35
Sexual interactions with someone over 18	3 (6.7)	10 (23.8)	Fisher's exact *p* = 0.04; OR = 4.4

### Adult victimization

As shown in Table 2, participants with ASC were 2.7 times more likely to endorse that they had experienced teasing during adulthood. There was a trend toward those with ASC being more likely to report sexual assault from a known adult, attempted or complete rape, and dating violence. Sexual victimization was further examined in order to separate contact victimization versus noncontact victimization. Sexual assault (by a known adult or unknown adult), and rape (attempted or completed) were summed (resulting in a score of 0–3). Individuals with ASC had significantly higher scores on this composite score than those without ASC (*U* = 1148.5, *p* = 0.03; ASC group *M* = 0.67, *SD* = 0.93; no ASC group *M* = 0.29, *SD* = 0.71) Participants without ASC were 4.4 times more likely to endorse assault with a weapon during adulthood. Participants with ASC did not report greater polyvictimization in adulthood than those without ASC (*U* = 894, *p* = 0.66; ASC group *M* = 6.16, *SD* = 5.52; no ASC *M* = 5.95, *SD* = 4.22).

**Table 2:** Frequency table for the 29 types of adulthood victimization on the modified JVQ-AR as reported by adults with and without ASC.

**Victimization Type**	**ASC *n* (%)**	**No ASC *n* (%)**	**Chi-square/Fisher's exact**
29 types of victimization, at least one type	41 (91.1)	39 (92.8)	χ2_(1)_ = 0.09, *p* = 0.77
Property Crime aggregate (at least one type)	25 (55.6)	28 (66.7)	χ2_(1)_ = 1.13, *p* = 0.29
Robbery	9 (20)	9 (21.4)	χ2_(1)_ = 0.03, *p* = 0.87
Theft	23 (51.1)	22 (52.4)	χ2_(1)_ = 0.01, *p* = 0.91
Vandalism	8 (17.8)	15 (35.7)	χ2_(1)_ = 3.59, *p* = 0.06; OR = 2.57
Physical Assault aggregate (at least one type)	27 (60)	25 (59.5)	χ2_(1)_ = 0.02, *p* = 0.96
Assault with a weapon	3 (6.7)	10 (23.8)	Fisher's exact *p* = 0.04; OR = 4.4
Assault without a weapon	20 (44.4)	16 (38.1)	χ2_(1)_ = 0.36, *p* = 0.55
Attempted assault	8 (17.8)	9 (21.4)	χ2_(1)_ = 0.18, *p* = 0.67
Kidnap, attempted or completed	0 (0)	2 (4.4)	Fisher's exact *p* = 1.00
Bias attack	2 (4.4)	2 (4.8)	Fisher's exact *p* = 1.00
Physical abuse	18 (40)	12 (28.6)	χ2_(1)_ = 1.26, *p* = 0.26
Assault by group or gang of peers	3 (6.7)	4 (9.5)	Fisher's exact *p* = 0.71
Genital assault	2 (4.4)	3 (7.1)	Fisher's exact *p* = 0.67
Dating violence	12 (26.7)	10 (23.8)	χ2_(1)_ = 0.09, *p* = 0.76
Maltreatment in Adulthood	29 (64.4)	21 (50)	χ2_(1)_ = 1.85, *p* = 0.17
Physical abuse	18 (40)	12 (28.6)	χ2_(1)_ = 1.26, *p* = 0.26
Psychological or emotional abuse	16 (38.1)	25 (55.6)	χ2_(1)_ = 2.66, *p* = 0.10; OR = 2.03
Peer/Coworker victimization aggregate (at least one type)	27 (60)	23 (54.8)	χ2_(1)_ = 0.24, *p* = 0.62
Assault by group or gang of peers	3 (6.7)	4 (9.5)	Fisher's exact *p* = 0.71
Genital assault	2 (4.4)	3 (7.1)	Fisher's exact *p* = 0.67
Bullying	12 (26.7)	11 (11.9)	Fisher's exact *p* = 0.11
Teasing, emotional bullying	27 (60)	15 (35.7)	χ2_(1)_ = 5.13, *p* = 0.02; OR = 2.7
Dating violence	12 (26.7)	10 (23.8)	χ2_(1)_ = 0.09, *p* = 0.09; OR = 1.16
Witnessed/indirect victimization aggregate (at least one type)	26 (57.8)	32 (76)	χ2_(1)_ = 3.31, *p* = 0.07
Witness domestic violence	4 (8.9)	4 (9.5)	Fisher's exact *p* = 1.00
Witness physical abuse	3 (6.7)	3 (7.3)	Fisher's exact *p* = 1.00
Witness assault with a weapon	6 (13.3)	10 (26.3)	χ2_(1)_ = 2.23, *p* = 0.14
Witness assault without a weapon	16 (35.6)	19 (46.3)	χ2_(1)_ = 1.03, *p* = 0.31
Household theft	10 (22.2)	15 (36.6)	χ2_(1)_ = 2.45, *p* = 0.14
Someone close murdered	5 (11.1)	2 (4.8)	Fisher's exact *p* = 0.44
Witness murder	3 (6.7)	3 (7.3)	Fisher's exact *p* = 1.00
Exposure to shooting, bombs, riots	6 (13.3)	12 (29.3)	χ2_(1)_ = 3.29, *p* = 0.07; OR = 2.69
Sexual victimization aggregate (endorsed at least one type)	21 (46.7)	17 (40.5)	χ2_(1)_ = 0.34, *p* = 0.56
Sexual assault, known adult	11 (24.4)	4 (9.5)	Fisher's exact *p* = 0.09; OR = 3.07
Sexual assault, unknown adult	6 (13.3)	3 (7.1)	Fisher's exact *p* = 0.49
Rape, attempted, or completed	13 (28.9)	5 (11.9)	Fisher's exact *p* = 0.07; OR = 3.01
Flashing or sexual exposure	8 (17.8)	14 (33.3)	χ2_(1)_ = 2.78, *p* = 0.10; OR = 2.31
Sexual harassment	12 (26.7)	8 (19.0)	χ2_(1)_ = 0.71, *p* = 0.40

### Adult perpetration

Table 3 presents the frequencies of endorsing each type and category of perpetration, and the comparisons across groups. No significant differences were found between groups on any form of perpetration, with very low rates reported. Groups did not differ on their polyperpetration score (*U* = 1006, *p* = 0.59, ASC group mean = 2.40, *SD* = 3.02; no ASC group *M* = 1.90, *SD* = 2.09).

**Table 3:** Frequency table for the 19 types of adulthood perpetration on the modified JVQ-AR as reported by adults with and without ASC.

**Victimization Type**	**ASC *n* (%)**	**No ASC *n* (%)**	**Chi-square/Fisher's exact**
19 types of perpetration, endorsed at least one type	32 (71)	25 (59.5)	χ2_(1)_ = 1.29, *p* = 0.26
Property Crime aggregate (at least one type)	25 (55.6)	28 (66.7)	χ2_(1)_ = 1.13, *p* = 0.29
Robbery	7 (15.6)	7 (16.7)	χ2_(1)_ = 0.02, *p* = 0.88
Theft	9 (20.5)	4 (9.5)	Fisher's exact *p* = 0.23
Vandalism	8 (19)	8 (18.2)	χ2_(1)_ = 0.01, *p* = 0.91
Physical Assault aggregate (at least one type)	27 (60)	25 (59.5)	χ2_(1)_ = 0.002, *p* = 0.96
Assault with a weapon	3 (6.8)	2 (4.8)	Fisher's exact *p* = 1.00
Assault without a weapon	14 (31.8)	15 (35.7)	χ2_(1)_ = 0.15, *p* = 0.70
Attempted assault	8 (18.2)	3 (7.1)	Fisher's exact *p* = 0.20
Kidnap, attempted or completed	0 (0)	0 (0)	–
Bias attack	0 (0)	0 (0)	–
Physical abuse of other adults	13 (29.5)	12 (28.6)	χ2_(1)_ = 0.01, *p* = 0.92
Committing assault with a group or gang of peers	1 (2.3)	1 (2.4)	Fisher's exact *p* = 1.00
Genital assault	3 (6.8)	2 (4.8)	Fisher's exact *p* = 1.00
Dating violence	5 (11.9)	10 (22.7)	Fisher's exact *p* = 0.26
Emotional abuse/bullying aggregate (at least one type)	19 (43.2)	16 (38.1)	χ2_(1)_ = 0.23, *p* = 0.63
Psychological or emotional abuse	19 (43.2)	15 (35.7)	χ2_(1)_ = 0.50, *p* = 0.48
Bullying	5 (11.4)	2 (4.8)	Fisher's exact *p* = 0.43
Sexual victimization aggregate (at least one type)	3 (7.1)	4 (9.1)	Fisher's exact *p* = 1.00
Sexual assault, known adult	1 (2.3)	0 (0)	Fisher's exact *p* = 1.00
Sexual assault, unknown adult	1 (2.3)	0 (0)	Fisher's exact *p* = 1.00
Rape, attempted, or completed	1 (2.3)	0 (0)	Fisher's exact *p* = 1.00
Flashing or sexual exposure	2 (4.5)	2 (4.8)	Fisher's exact *p* = 1.00
Sexual harassment	3 (6.8)	2 (4.8)	Fisher's exact *p* = 1.00

### Mediators of victimization and perpetration

As expected, the ASC group reported less developed sociocommunicative competence (ASC *M* = 3.32, *SD* = 0.40; no ASC *M* = 4.05, *SD* = 0.40; *U* = 200.00, *p* < 0.001) and poorer emotion regulation abilities (ASC *M* = 2.72, *SD* = 0.57; no ASC *M* = 1.88, *SD* = 0.51; *U* = 200.00, *p* < 0.001) compared to the comparison group. Neither sociocommunicative competence or emotion regulation were significantly correlated with childhood polyvictimization or adult polyvictimization in the ASC group or the non ASC group (all *p*'s > 0.05). Multiple mediation analyses were used in order to further examine whether emotion regulation and sociocommunicative competence were related to the group differences found in childhood polyvictimization experiences. Table 4 shows the unstandardized coefficients of each pathway, the confidence intervals, and the bootstrapping results based on 1,000 resamples. The total direct effect (path c) of ASC status approached significance before entering the mediator variables, *z* = 1.95, *p* = 0.05. The relationship between ASC diagnosis and polyvictimization in childhood was not mediated by sociocommunicative status or emotion regulation. The direction of estimates in the mediator pathways (path a) indicated that having ASC was associated with lower sociocommunicative competence (*t* = −8.30, *p* < 0.001), and poorer emotion regulation (*t* = 7.27, *p* < 0.001). The total indirect effects did not suggest the presence of mediation, as emotion regulation and sociocommunicative competence were not related to polyvictimization (path b). Mediation analyses were not computed for adult polyvictimization or polyperpetration, as no significant differences were found between groups.

**Table 4:** Multiple mediation analysis results for the mediating effect of sociocommunicative competence and emotion regulation on the relationship between group and childhood polyvictimization after controlling for sex and age.

				**Bootstrapping for Indirect Results**				
				**Point estimate**		**95% CI**		
**IV, Mediators, and Control**	**Path**	***B***	***SE***	***z/t***	***p***		**Lower**	**Upper**
Sex	Control	0.81	1.41	0.57	0.57			
Age	Control	0.08	0.08	1.03	0.30			
Group	C	2.68	1.37	1.95	0.05	−0.17	−3.9	4.55
	C'	2.84	2.01	1.42	0.16			
Emotion Regulation	A	0.85	0.12	7.27	<0.001	1.17	−1.95	3.88
	B	1.37	1.36	1.01	0.32			
Sociocommunicative Competence	A	−0.77	0.09	−8.30	<0.001	−1.34	−4.05	2.12
	B	1.73	1.71	1.01	0.32			

## Discussion

### Victimization

This is the first study to explore self-reported experiences of many forms of victimization and perpetration in adults with ASC compared to those without. ~90% of participants with and without ASC reported experiencing at least one form of victimization in childhood, and approximately the same number reported victimization in adulthood. Using the same measure of childhood victimization, other research has found that 97% of college age women (48) and 80% of young adult men and women who had been identified as “at risk for high school drop out” endorse experiencing at least one form of victimization in childhood (36). It appears that using a broad measure of violence experiences, in a broader range of adult ages, results in similar high rates.

Adults with ASC reported a greater breadth of victimization during childhood compared to adults without ASC, matched on sex, IQ, and age. Adults with ASC were more likely to report that as a child, they experienced physical abuse, psychological/emotional abuse from an adult, peer/sibling victimization, various forms of bullying from peers, robbery, and sexual assault by a peer than respondents without ASC. The current research also shows that they are at risk for violence victimization more broadly in childhood. Although the short and long-term impact of victimization, or trauma more broadly, on individuals with ASC is relatively unknown, peer victimization in youth with ASC has been related to internalizing and externalizing symptoms (6, 49), and maltreatment among youth with ASC has been related to externalizing behavior, suicide attempts, conduct and academic problems (7). It is important that childhood victimization in various contexts (home, school, and community) be addressed in order to keep this vulnerable group of youth safe. There is emerging evidence for strength-based school programming to reduce experiences of victimization in general (50), and these programs could be examined for their utility in decreasing victimization for those with ASC.

No differences were found between groups on polyvictimization in adulthood, though differences did emerge in specific kinds. Individuals with ASC were more likely to report experiencing teasing/emotional bullying from other adults, which speaks to a continued risk for interpersonal difficulties with peers across the lifespan. Adults with ASC, whether in the role as an employee or with peers in the community, may benefit from specific training on what constitutes bullying and harassment and how to effectively manage those situations (51). Adults with ASC were also more likely to endorse experiencing some form of sexual victimization that involved contact, including sexual assault and rape, in line with previous research (5). There has been some research advocating for interventions targeting the risk of sexual victimization of individuals with developmental disabilities (52), focusing often on addressing self-protection and assertiveness [e.g., (53)], and education on sexual abuse for support workers (54). In considering how to best reduce the risk of interpersonal violence victimization for adults with ASC, proactive and accessible programming that promotes inclusion and healthy relationships within relevant contexts (including the home, school, workplace, and community levels) are needed.

### Perpetration

Groups had similar rates across all forms of perpetration, categories of perpetration, and on polyperpetration, largely due to the equally low endorsements. Low rates were found for both severe and more minor occurrences of violence perpetration. These results map onto the existing reviews finding low rates of perpetration in individuals with ASC and no clear association with violent crime (15, 16). While other studies have examined inpatients, file reviews of incarcerated individuals, or parent/caregiver report, the current study is the first to compare two matched community samples. Researchers and clinicians have cautioned that the sensational and unusual nature of some criminal incidents with individuals with ASC may garner media attention, and perpetuate the notion that individuals with ASC are more violent that individuals without ASC, which is not the case (55). It may be the case that perpetrators with ASC present differently than perpetrators without ASC, with authors describing the links between the symptomology of ASC and offending behaviors (56). These differences will not emerge in examining rates *per se*, but in the nuances of how perpetration is expressed and the contexts that underlie these behaviors.

### Mediators of victimization

Contrary to expectations, sociocommunicative ability and emotion regulation deficits in adults with ASC did not explain a heightened risk for victimization. In fact, neither polyvictimization in childhood or adulthood was correlated with either variable, in either group. In the typical population, many additional factors have been associated with discrete types of victimization (e.g., bullying) and with overall risk, including age, gender, childhood experiences of victimization (emotional/physical/sexual abuse), and mental and physical health problems [e.g., (57–62)], which could be examined in future research. As well, models of victimization largely underscore the important of context, and the dynamics among individual and contextual factors (13). Researchers have begun to study the interplay, and differential impact, of individual and contextual factors, and some have found that contextual factors, such as dangerous neighborhoods, play an important role in adult repeat victimization (63). This study did not consider contextual risk factors for interpersonal violence (e.g., SES, education, family relationships etc.), which may provide a more comprehensive understanding of polyvictimization experiences.

### Limitations and future directions

The present study is based on retrospective reporting, which limits any discussion of causality and directionality. Longitudinal design could be used to further examine the pathways that lead to violence victimization and perpetration. Participation was not anonymous, questions were answered in the presence of a researcher, and we did not measure social desirability, making it difficult to know whether participants in either group were under reporting their experiences. We also did not attempt to substantiate reports with other informants, as we sought to understand and value self-reported experiences. Future research could examine both self- and informant-report to examine how responses may be correlated. It is possible that this sample represents a more well-adjusted and functional group of individuals with ASC, and it is unclear whether these results generalize to those who have greater difficulties, as the link between level of functioning and the violence experiences of those with ASC is not well understood. We also did not employ the ADOS-2 to ensure that the comparison group did not have significant symptoms of ASC, though none reported identifying as on the spectrum or being diagnosed with ASC.

This study has both statistical and psychometric limitations. This study was aimed to describe different kinds of victimization and perpetration, and was the first study to apply the JVQ-AR with an adult focus and with respondents with ASC. Alternative measures of violence that are psychometrically validated could provide different results, and are an important endeavor given the current pattern of reported polyvictimization. Additionally, our study had a small sample size and relatively low power for low frequency occurring kinds of victimization or perpetration. There multiple exploratory comparisons do increase the risk of Type I error, and we did not correct for this as a result of the exploratory nature of these comparisons and the relatively small, but important, clinical sample. This remains an important first step to inform future investigations. Finally, this sample of participants had proportionally more women than expected in ASC research, and it is likely that this does not reflect the gender distribution in the population. While the two groups were matched on gender, education level, ethnicity status, age, and IQ, we did not collect or match on other demographics which may differ between groups or be associated with victimization (e.g., employment status, poverty).

## Conclusion

Participants with ASC are at considerable risk for experiencing polyvictimization in childhood and for bullying and sexual contact victimization in adulthood. This increased vulnerability to victimization, especially in childhood, highlights the need for intervention and proactive prevention strategies to decrease vulnerability and impact. These findings have serious implications for how we discuss violence victimization, and suggest that understanding interpersonal violence more broadly is critical to ensuring that we identify and target factors that may place people with ASC at risk for many kinds of negative experience.

## Ethics statement

This study was carried out in accordance with the recommendations of the Canadian Tri-Council Policy Statement Ethical Conduct for Research Involving Human Participants, and the York University Senate Policy, Research Involving Human Participants. The protocol was approved by the York University Human Participants Review (Ethics) Sub-Committee of the Office of Research Ethics. All subjects gave written informed consent in accordance with the Declaration of Helsinki.

## Author contributions

MF and JW conceptualized the study, analyzed the data, and contributed to manuscript preparation; MF conducted the recruitment and data collection. All authors agree to be accountable for the content of the work.

### Conflict of interest statement

The authors declare that the research was conducted in the absence of any commercial or financial relationships that could be construed as a potential conflict of interest.
